# Associations between the built environment, total, recreational, and transit-related physical activity

**DOI:** 10.1186/1471-2458-14-693

**Published:** 2014-07-07

**Authors:** Eric de Sa, Chris I Ardern

**Affiliations:** 1 School of Kinesiology and Health Science, York University, Toronto M3J1P3, ON, Canada

**Keywords:** Physical activity, Built environment, Hierarchical linear modeling, GIS, CCHS

## Abstract

**Background:**

Many aspects of the built, physical environment have been shown to be associated with physical activity, but little research has focused on the unique circumstances and urban form of the suburban environment. The following analyses explore the associations between features of the built environment and components of overall physical activity, after accounting for neighborhood variability using hierarchical linear modeling.

**Methods:**

These analyses utilized regionally-specific Geographic Information Systems data along with health measures collected from the 2007–8 Canadian Community Health Survey. Linear and logistic regression models explored the associations between measures of the built environment with leisure-time and transport-related physical activity.

**Results:**

Respondents living with the highest number of intersections were more likely to engage in walking or cycling for leisure (OR: 1.85 CI 95%: 1.23-2.78), and in general, those living in areas with higher residential density were more likely to engage in active modes of transportation (OR: 2.67, CI 95%: 1.34-5.34).

**Conclusions:**

Further analyses are necessary to clarify the extent to which modifications to such features of the built environment may improve physical activity participation in similar suburban communities.

## Background

The environment in which we live is a major determinant on overall health and well-being [1]. Ecologic models acknowledge that there are individual and community-level factors that may influence health behaviours [2]. While the pathways to health and disease may be both direct and indirect, when taken together, emerging evidence suggests that patterns of discretionary physical activity (PA) participation may be significantly influenced by those features of the physical and social environment associated with PA accessibility [3,4]. In order for the inactive portion of our society to effectively shift towards one that is more active, strategies for PA promotion must be targeted beyond individual behavior change to incorporate strategies that engage whole health organizations, institutions, and communities.

Current estimates suggest that between ~3% and 28% of the variance in PA participation can be accounted for by neighbourhood-level differences in the built environment [5,6], consistent with ecologic models which view PA as having multiple influences; including intrapersonal, interpersonal, policy, and environmental components [7]. Moreover, research suggests that the role of the built environment in influencing opportunities to engage in PA varies [8], as neighborhoods may provide constraints and opportunities for different forms of PA [9]. While many vigorous forms of PA are engaged in for recreational or health-related purposes, walking and cycling remain two of the most common forms of PA [10] and can be done for leisure, recreation, exercise, occupational, or transport-related reasons. In turn, this may make walking and bicycling more susceptible to environmental influences [11]. In addition to studying leisure-time PA, transport-related activities, such as walking or cycling, are alternate means to increase total daily energy expenditure [12].

While a number of studies have assessed the impact that both individual-level and neighborhood-level characteristics have on the frequency, duration, and engagement in PA [5,6,13,14], this work has largely been limited to assessing walking behaviour [5,6], or used multiple data sources to additional questionnaires to obtain demographic and PA data [13,14]. Moreover, little work has been done within the Canadian context with demographic and PA information obtained during the same question period. Therefore, the aim of the current analysis is to quantify the association between different measures of the built environment with leisure-time and transport-related PA.

## Methods

### The Canadian Community Health Survey (CCHS) 2007–2008

This analysis used data from the 2007–2008 Canadian Community Health Survey (CCHS 2007–2008, master data file; Statistics Canada, Health Statistics Division and Special Surveys Division), obtained through the limited data access program at the York University chapter of the Toronto Research Data Center of Statistics Canada. The CCHS is a cross-sectional survey that collects information on health status, health care utilization, and health determinants. Reliable estimates at the health region level are obtained by sampling a large number of respondents throughout Canada. To give equal importance to the health regions in each province, a multi-stage sample allocation strategy was employed.

The CCHS questions are designed for computer-assisted interviewing (CAI). Approximately 130 000 persons across 121 health regions were sampled during the data collection period from January 2007 to December 2008 inclusive. Three sampling frames were used to select the sample of households: 49% of respondents were obtained from an area frame, 50% from a list frame of telephone numbers, and the remaining 1% from random digit dialing. Interviews were conducted both in person and over the telephone. To reduce the number of errors in survey reporting, the CAI is not programmed to accept out-of-range values and flow errors are controlled through programmed skip patterns. For inconsistent or unusual reporting, warning messages are invoked and further edits are performed at the Head Office during the data processing step.

### The Regional Municipality of York (York Region)

The Municipality of York Region is located directly north of Toronto and comprises nine municipalities: City of Markham, City of Vaughan, Town of Richmond Hill, Town of Aurora, Town of Newmarket, Township of King, Town of Whitchurch-Stouffville, Town of East Gwillimbury, and Town of Georgina. The three municipalities closest to Toronto have the highest population growth rates (Vaughan, Markham, and Richmond Hill). During the period of 1996–2001, York Region was the fastest growing Census Division in Canada with 30% of the population identifying themselves as visible minorities [15]. By 2010, the total population had exceeded one million people. From 1996 to 2001, there was a 30% increase in the employment labour force from 297,600 to 387,700. As of 2007, an estimated 485,000 people worked in York Region and is projected to increase to 800,000 jobs by 2031 [16]. With the forecasted high rate of population and employment growth, York Region provides a unique opportunity to look at a demographically diverse population found outside of a major metropolitan area such as Toronto and to explore associations between the built environment and PA participation.

### Exclusion criteria

All Canadians age 12 y and older were considered eligible for participation in the CCHS study (with few exceptions including individuals living on Indian Reserves or Crown Lands, institutional residents, full-time members of the Canadian Forces, and residents of certain remote regions). All respondents who were unable to be properly geocoded with their corresponding postal-code address or whose address fell outside the York Region boundary were eliminated from analysis. Those respondents that were located within the York Region boundary belonged to one of the nine municipalities. These analyses included respondents 18 years or older.

### Dependent variables (Physical Activity)

For continuous outcomes, daily minutes engaged in walking or cycling for leisure was calculated based on the frequency of engaging in walking or cycling within the past 3 months and the average daily duration spent in the activity. Leisure-time daily energy expenditure (LTDEE) (kcal/kg/day (KKD)) spent in all leisure time activities was a derived variable previously calculated for each respondent by Statistics Canada. For dichotomous outcomes, respondents were classified as having engaged in walking or cycling for leisure-time purposes (any/none) and walking or cycling for transport-related purposes (any/none).

General measures of PA included both a leisure-time physical PA (LTPA) index and an index with transport-related PA (TRPA) and LTPA combined. The resulting average daily energy expenditure was used to classify participants as: inactive (<1.5 kcal/kg/day; KKD), moderately active (1.5-2.9 KKD), and sufficiently active (≥3.0 KKD). The general measure of leisure-time PA asked respondents about their activity patterns within the past 3 months, including (but not limited to) the following activities: walking for exercise, gardening or yard work, swimming, bicycling, popular or social dance, home exercises, ice hockey, ice skating, in-line skating or rollerblading, jogging or running, golfing, exercise class or aerobics, downhill skiing or snowboarding, bowling, baseball or softball, tennis, weight-training, fishing, volleyball, basketball, soccer, and any other self-described form of PA participation. In addition, respondents were asked frequency and duration of both walking and cycling to school or work (transport-related physical activity).

### Geographic Information Systems (GIS) Software

ArcView GIS, version 9.3 software [17] was used to geocode participants by postal-code address to existing maps in the CanMap StreetFiles: Ontario and Platinum Postal Code Suite (both are products from DMTI Spatial Corporation). The postal code polygons within the shape file differed in size depending on the area each represented. Respondents would normally be located on the periphery of each polygon with the inclusion of specific address information relating to street and house/unit number; however, this data was not available within the CCHS, resulting in the geocoding of respondents to the centroid for all analyses. This strategy would create a greater displacement from the periphery to the centroid for respondents belonging to postal code regions that covered larger (compared to smaller) areas of land.

A series of map layers specific to each built environment measure (including: residential density, area of building space, area of parks/green spaces, and intersections) were used to quantify the characteristics within a 500 m buffer zone. The geocoding process resulted in the formation of a centroid to represent each 6-digit postal code region. Once data relating to the built environment measures were collated for each participant, the spatial data was quantified and exported into a SAS compatible database that was linked with the PA and individual-level covariates for each participant.

### Independent variables (Built Environment Measures)

All built/neighborhood environment measures were quantified within a 500 m buffer zone around the centroid of each postal code address. A buffer region of 500 m was chosen as it can be approximated to walking for 5 minutes, suggesting that walking to and from the periphery would equate to engaging in 10 minutes of walking activity and could be considered one small bout of the recommended daily level of PA participation [18,19].

### Building area, parks/green space area

The total area occupied within the 500 m buffer is 78.53975 hectares (1 hectare (ha) = 10 000 m^2^). Total area of the buffer zone occupied by building area (first-storey landscape), and total area of parks/green space were calculated around each centroid. Parks/Green space was calculated using two methods: i) both public (parks, provincial parks, sports fields) and private (golf courses, driving ranges, amusement parks, historical sites, exhibition grounds) areas; and ii) public areas only.

### Residential density

Residential density was ascertained by calculating the number of dwellings (detached, semi-detached, condos, and apartments) and dividing by the total area of the buffer zone (units/hectare).

### Intersections

Number of street intersections including those with traffic lights and those without (excluding freeway ramps) were counted within each buffer zone.

### Covariates

Self-reported weight and height were used to classify Body Mass Index (BMI: weight (kg) / height^2^ (m^2^). Educational attainment (<high school, high school, some post-secondary, and completed a post secondary degree/diploma), income tertiles (≤ $59 999, $60 000-$99 000, ≥ $100 000), ethnicity (white, non-white), smoking status (non-smoker, former smoker, occasional smoker, and daily smoker), age, and sex were also treated as covariates.

### Statistical analysis

The association between each built environment measure and PA type (LTPA and TRPA) were examined using multilevel regression models. Using hierarchical linear modeling (HLM), both neighborhoods and residents are treated as units of analyses allowing evaluation of between neighbourhood and within neighbourhood variability in the different PA outcomes [6], a distinct advantage to traditional methods of cluster analysis in health. Model 1 estimated the univariate association between the built environment measure and PA outcome, and Model 2 adjusted for all other covariates (multivariable model). All continuous outcomes (daily minutes spent in leisure-time walking and cycling pursuits and daily energy expenditure for all leisure time activities) were analysed using linear regressions (PROC MIXED), while dichotomous outcomes were analysed using logistic regressions (PROC GLIMMIX). All statistical analyses were conducted using SAS version 9.2 [20] with statistical significance set at alpha <0.05. After removing respondents with missing data, some respondents were unable to be properly geocoded and were not included in the analyses (N = 48). Removal of these respondents was a limitation of the GIS maps used to geocoded, and the impact on population-weights could not be assessed. As such, population weights were not applied to the multivariable models.

## Results

The demographic characteristics of the respondents and characteristics of the built environment are presented in Table 1. A total of 1 158 respondents were included in the analyses (μ_age_ = 47.9 y; μ_BMI_ = 25.6 kg · m^-2^, and; 49.6% female). In general, the majority of the sample was white (69.6%) and well-educated (80% with at least post-secondary education). Nearly half of the sample had never smoked (44.8%) and were either moderately or sufficiently active (47.7%).

**Table 1:** Demographic and local built environment characteristics for respondents belonging to the York Region health unit

**Demographics (n = 1 158)**		
		**Mean (SD)**
Age	Years	47.9 (16.9)
BMI	kg/m^2^	25.6 (4.4)
		%
Sex	Females	49.6
Ethnicity	White	69.6
Education	Post-Secondary	80.0
Smoker	Never Smoked	44.8
**Local Built Environment (500m)**		
**Built Environment Measure**		**Mean (SD)**
Building Area (hectares)		1.5 (1.9)
All Green Space (hectares)		6.0 (9.9)
Public Green Space Only (hectares)		4.9 (9.2)
Residential Density (units/hectare)		6.8 (4.4)
		**Range**
Intersections		0 - 133

Note: 1 hectare = 10 000 square metres; Income was classified into tertile ranges (lowest: ≤ $59 999, middle: $60 000-$99 000, highest: ≥ $100 000).

Table 2 gives an overview of the outcome variables. Respondents spent an average of 17.7 minutes/day either walking or cycling for leisure and their median daily energy expenditure was 1.3 KKD for leisure-time pursuits. Overall, 69.8% of respondents participated in any form of walking or cycling for leisure and 14.4% engaged in any form of walking or cycling for transport-related pursuits. The PA variable was not transformed since the continuous KKD was calculated based on categorized approximations for the amount of time spent in certain activities and was not originally assessed on a continuous scale. Furthermore, it would be more difficult to apply the results from a transformed-PA variable to real-world applications.

**Table 2:** Outcome characteristics for respondents belonging to the York Region health unit

**Outcomes**	**Mean (SD)**
Walking/Cycling for Leisure (minutes/day)	17.7 (18.0)
Leisure-Time Daily Energy Expenditure (KKD)	1.9 (2.2)
	N (%)
Any Walking/Cycling for Leisure	808 (69.8)
Any Walking/Cycling for Transportation	126 (14.4)

### Walking / cycling for leisure-time PA (minutes/day)

In multivariate analysis, no single measure of the built environment was found to be significantly associated with walking or cycling for leisure-time PA (Table 3).

**Table 3:** Association of built environment measures with total time spent walking or cycling for leisure-time activities (minutes / day) and with daily energy expenditure (KKD) for leisure-time activities (LTDEE)

**Built Environment**	**Model 1†**		**Model 2‡**	
**Measure**	**β**	**(SE)**	**β**	**(SE)**
*Walking/Cycling for leisure-time activities (minutes / day)*				
Building Area	-0.128	(0.398)	-0.311	(0.394)
All Green Space	-0.057	(0.073)	-0.066	(0.071)
Public Green Space	0.009	(0.078)	-0.005	(0.077)
Residential Density	0.070	(0.166)	0.042	(0.164)
Intersections	0.054	(0.028)	0.049	(0.028)
*Leisure-time daily energy expenditure (KKD)*				
Building Area	-0.106**	(0.036)	-0.071*	(0.035)
All Green Space	0.004	(0.007)	0.005	(0.007)
Public Green Space	0.006	(0.007)	0.008	(0.007)
Residential Density	-0.038*	(0.016)	-0.026	(0.016)
Intersections	0.001	(0.003)	0.003	(0.003)

†model 1: unadjusted.

‡model 2: adjusted for the following covariates: age, sex, bmi, ethnicity, income, education, smoking status.

*, p < 0.05.

**, p < 0.005.

### Leisure-Time Daily Energy Expenditure (LTDEE) (KKD)

**Figure 1:**
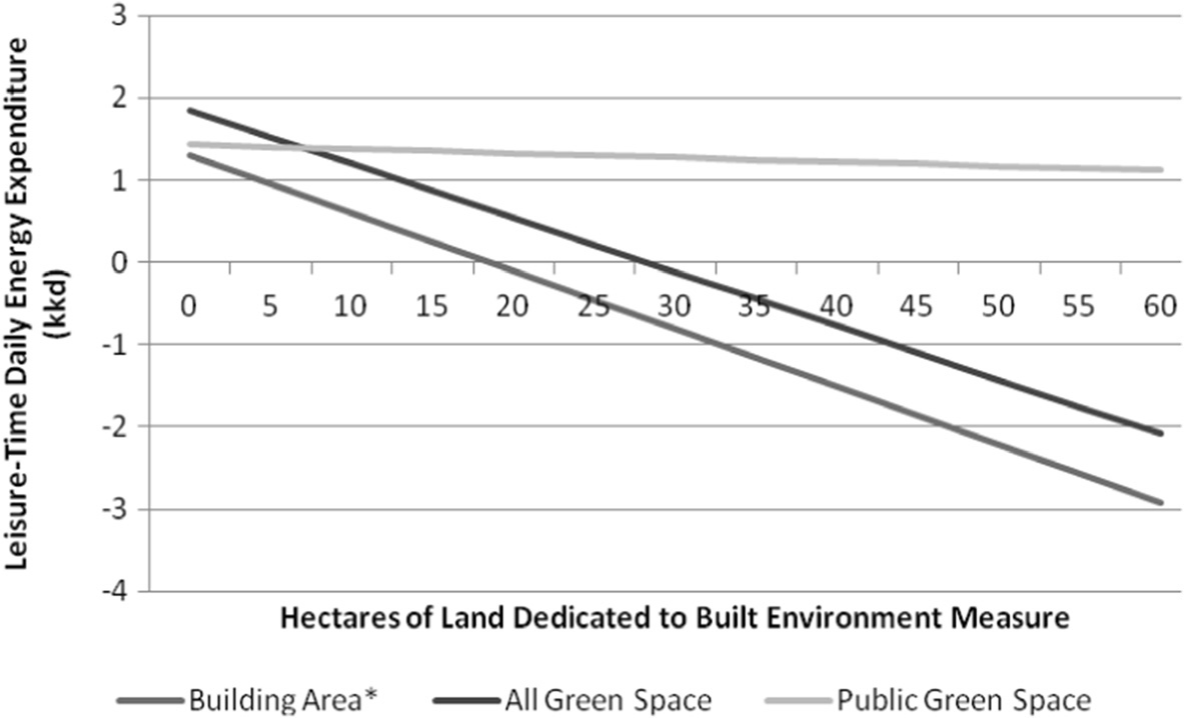
Association of built environment measures with daily energy expenditure (KKD) for leisure-time activities (LTDEE).

Although higher residential density was associated with a decrease in LTDEE (β = -0.038, p < 0.05), this association did not remain significant within the fully adjusted model (Table 3), whereas an increase in building area was negatively associated with LTDEE (Model 1, β = -0.106, p < 0.005; Model 2, β = -0.071, p < 0.05) (Figure 1).

### Any walking or cycling for leisure

Compared to respondents living with the fewest intersections within their 500 m buffer zone (quartile 1), those living in the highest quartile were more likely to engage in walking or cycling for leisure (OR: 1.85 CI 95%: 1.23-2.78), even after adjustment for covariates (Table 4).

**Table 4:** Association of built environment measures with engaging in any form of walking or cycling for leisure-time in 500 m buffer zone

		**Model 1†**		**Model 2‡**	
**BEM**		**OR**	**CI**	**OR**	**CI**
Building Area	Q1	1.00	(ref)	1.00	(ref)
	Q2	1.38 [0.92 - 2.06]		1.42 [0.94 - 2.15]	
	Q3	1.28 [0.85 - 1.93]		1.43 [0.94 - 2.18]	
	Q4	0.81 [0.54 - 1.22]		0.92 [0.60 - 1.41]	
All Green Space	Q1	1.00	(ref)	1.00	(ref)
(public + private)	Q2	0.96 [0.64 - 1.42]		1.04 [0.69 - 1.56]	
	Q3	1.06 [0.73 - 1.53]		1.18 [0.80 - 1.73]	
	Q4	1.11 [0.76 - 1.61]		1.23 [0.83 - 1.82]	
Public Green Space	Q1	1.00	(ref)	1.00	(ref)
	Q2	0.87 [0.57 - 1.34]		1.00 [0.64 - 1.56]	
	Q3	1.15 [0.80 - 1.65]		1.25 [0.86 - 1.82]	
	Q4	1.01 [0.70 - 1.44]		1.12 [0.77 - 1.63]	
Residential Density	Q1	1.00	(ref)	1.00	(ref)
	Q2	1.28 [0.86 - 1.92]		1.40 [0.92 - 2.12]	
	Q3	1.03 [0.69 - 1.55]		1.13 [0.74 - 1.73]	
	Q4	1.04 [0.68 - 1.58]		1.19 [0.77 - 1.86]	
Intersections	Q1	1.00	(ref)	1.00	(ref)
	Q2	1.31 [0.90 - 1.91]		1.41 [0.95 - 2.09]	
	Q3	1.14 [0.78 - 1.68]		1.28 [0.86 - 1.90]	
	Q4	1.65* [1.11 - 2.46]		1.85* [1.23 - 2.78]	

†model 1: unadjusted.

‡model 2: adjusted for the following covariates: age, sex, bmi, ethnicity, income, education, smoking status.

*,p < 0.05.

### Any walking or cycling for transport

Respondents living in the fourth quartile (OR: 2.67, CI 95%: 1.34-5.34) of residential density and the second quartile (OR: 2.39, CI 95%: 1.25-4.56) of intersections were more likely to engage in active modes of transportation (such as walking or cycling to work or school) compared to the first quartiles of the respective built environment measures (Table 5).

**Table 5:** Association of built environment measures with engaging in any form of walking or cycling for transportation in 500 m buffer zone

		**Model 1†**		**Model 2‡**	
**BEM**		**OR**	**CI**	**OR**	**CI**
Building Area	Q1	1.00	(ref)	1.00	(ref)
	Q2	1.71 [0.93 - 3.15]		1.80 [0.94 - 3.42]	
	Q3	1.63 [0.87- 3.03]		1.83 [0.95 - 3.55]	
	Q4	1.53 [0.81 - 2.89]		1.93 [0.97 - 3.83]	
All Green Space	Q1	1.00	(ref)	1.00	(ref)
(public + private)	Q2	0.92 [0.50 - 1.71]		0.95 [0.49 - 1.84]	
	Q3	1.32 [0.76 - 2.30]		1.50 [0.83 - 2.71]	
	Q4	1.50 [0.86 - 2.61]		1.36 [0.75 - 2.46]	
Public Green Space	Q1	1.00	(ref)	1.00	(ref)
	Q2	0.98 [0.50 - 1.91]		1.07 [0.52 - 2.20]	
	Q3	1.14 [0.67 - 1.97]		1.24 [0.69 - 2.23]	
	Q4	1.52 [0.89 - 2.58]		1.51 [0.85 - 2.68]	
Residential Density	Q1	1.00	(ref)	1.00	(ref)
	Q2	1.78 [0.94 - 3.36]		1.87 [0.94 - 3.71]	
	Q3	1.60 [0.83 - 3.07]		1.99 [0.98 - 4.05]	
	Q4	2.50* [1.33 - 4.69]		2.67* [1.34 - 5.34]	
Intersections	Q1	1.00	(ref)	1.00	(ref)
	Q2	2.70* [1.48 - 4.95]		2.39* [1.25 - 4.56]	
	Q3	1.74 [0.91 - 3.32]		1.70 [0.86 - 3.38]	
	Q4	1.42 [0.73 - 2.77]		1.28 [0.63 - 2.60]	

†model 1: unadjusted.

‡model 2: adjusted for the following covariates: age, sex, BMI, ethnicity, income, education, smoking status.

*, p < 0.05.

## Discussion

It has been recommended that people who are physically inactive should start with short sessions (5–10 minutes) of PA before building-up to longer durations of activity [18], and even walking at a brisk pace for 5 (~500 m) [13,14,21] or 10 minutes (~1 km) may be associated with significant health benefits [18]. In a buffer zone of 500 m fixed at the centroid of the respondent’s postal code address, higher residential density and intersection frequency was associated with greater odds of engaging in walking or cycling for transportation purposes, whereas intersection frequency was associated with walking or cycling for leisure-time. Consistent with previous literature [5,6,11] hectares of building area were found to be negatively associated with energy expenditure. Although some previous studies have also demonstrated significant associations between walking and bicycling for both leisure-time and commuting purposes with area of green and recreational space [6,14], no significant associations were found with either parks/green space measure and any form of walking or cycling for leisure-time or transport-related purposes.

These previous results notwithstanding, a greater amount of land dedicated to building area was associated with lower overall LTDEE, a finding that may be accounted for in part by an increase in sensitivity of the retail measure to identify pedestrian activity. Previous research calculated a ratio of the retail building floor area to the retail land floor area footprint with the rationale that a low ratio would indicate more parking, and a high ratio would indicate less surface parking and fewer setbacks which in turn promotes pedestrian activity [22]. However, the current analyses could not distinguish for what purpose the building area was designated, nor could a ratio be calculated, as only ground-level area footprint data was available. Therefore, the finding that higher levels of building area was related to lower levels of LTDEE may suggest that building area impacts the frequency or duration that respondents can engage in leisure-time PA. This may be possible if we assume higher levels of building area also require more space dedicated to parking or streets dedicated to vehicle traffic without accounting for pedestrian activity. While the significant association between building area and LTDEE was small, the results suggest that increasing the area within a local neighborhood dedicated to building space may hinder PA levels. However, without taking into account destinations to travel or a building footprint ration, further research is necessary to clarify the association between building area and LTDEE.

National health surveillance surveys have relied on sampling strategies that typically draw data from administrative boundaries or sociodemographic variables, thereby disregarding variations in distribution of built environment variables. Using spatial sampling techniques over neighborhood-based methods may require smaller sample sizes, be more cost-effective, and may be more generalizable to the broader population since spatial sampling does not impose a pre-specified neighborhood from which to draw a study sample [3]. Therefore, a major strength of the current analyses was the use of a population-based sample of respondents from the CCHS; however, in order to more fully characterize these relationships, future studies would benefit from exploring interactions between age, sex, socioeconomic, and ethnic subgroups to better understand factors that may account for additional neighbourhood-level variation in PA. Previous research has shown that rates of walking and cycling of children to school are inversely related to socioeconomic status [23,24]. Understanding features of the built environment that either promote or constrain PA may assist in closing the gap in patterns of PA participation in traditionally marginalized groups. Finally, the CCHS sampled respondents in all 9 municipalities of York Region during each month of the 2-year collection cycle. Although this mode of sampling is unlikely to impact on the spatial relationships observed here, future research would benefit by taking into account the month of data collection to account for seasonal effects on physical activity.

Several limitations of this study also warrant mention. First, while these analyses included a diverse age range and the sampling strategy incorporated a representative socioeconomic sample of the York Region population, it remains unclear whether the observed associations are similar across all subgroups of the population. Therefore, further research is necessary to examine whether different populations experience the same built environment differently (i.e. do subpopulations living in the same neighborhood and sharing the same space interact with the built environment distinctly from one another?). Due to sample size restrictions it was not possible to explore these interactions in the present analysis. Second, since these analyses were conducted using cross-sectional survey data, causality cannot be inferred. However, when taken together, they highlight the need for additional multilevel analyses to confirm the associations between physical activity and the built environment within rapidly growing, diverse regions of Canada. By combining multiple CCHS survey cycles, a clearer understanding of subgroup variation may be possible. While this approach (with different respondents for each survey cycle) could not be interpreted as longitudinal in design, when repeated in other regions in Canada, it could provide period estimates that could provide important insight for exploring changes in physical activity with changes in neighborhood landscape. Although the mode of sampling is unlikely to impact on the spatial relationships observed here, future research would benefit by taking into account the month of data collection to account for seasonal effects on physical activity. In addition, as with all survey data, social desirability bias should be acknowledged. Finally, it is important to note that respondents are not asked the reason for selecting their current address as the question is not addressed during the CCHS interview process. It is unclear if the resident has selected their household for reasons based on neighborhood safety, proximity to schools, traffic flow, socio-cultural factors, aesthetics, or a variety/combination of many other factors that relate to homeownership and neighborhood dwelling. While perceived neighbourhood safety has been found to be associated with physical activity [4,25-27], it could not be assessed within these analyses.

Future work should explore expanding the buffer zone to cover a larger walking distance provided sufficient data can be obtained to incorporate more measures of the built environment, thus providing a more robust estimation of the local landscape. It is possible that the magnitude of effects found within these analyses would differ depending on the size of the buffer zone. It would be helpful to assess points of interest in regard to walking distance within the local neighborhood. Researchers investigating associations between leisure- and transport-related physical activity with places to commute would benefit from calculating buffer zones based on network distance as this provides a more accurate indication of the routes realistically travelled. Road network data containing information on sidewalks and park trails/paths would help define pathways that would be exclusively used by cars and those that could be used for pedestrian and cyclists.

## Conclusion

The current analyses found that living in the highest regions of intersections and residential density was associated with higher levels of LTPA and TRPA, respectively, and a modest association between number of intersections within one’s neighbourhood and the likelihood that they engaged in walking/cycling for transport-related activities. Taken together, this suggests that respondents in York Region living in areas similar to major urban centres that have a greater number of intersections (increased street connectivity) and a higher level of residential density were more likely to engage in PA. While neighborhood level covariates could not assessed, these analyses still demonstrate the need to consider variability between local neighborhoods when exploring the associations of built environment measures and PA. In particular, these analyses found that certain features of the built environment (i.e. residential density, intersections, and building area) were independently associated with PA behaviours; however, while no associations were found with the amount of green space within a 500 m buffer zone. Further research is necessary to clarify the extent to which modifications to such features of the built environment may improve physical activity participation in similar suburban communities.

## Competing interests

The authors declare that they have no competing interests.

## Authors’ contributions

Contributions to the final manuscript are as follows: EdS and CA applied for and acquired the data. EdS and CA conceived of the design and analysis. EdS carried out the statistical analysis. EdS drafted the initial manuscript. Critical review, editing, and interpretation of the results were conducted by EdS and CA. Both authors read and approved the final manuscript.

## Pre-publication history

The pre-publication history for this paper can be accessed here:

http://www.biomedcentral.com/1471-2458/14/693/prepub
